# Perceptions of lifestyle-related risk communication in patients with breast and colorectal cancer: a qualitative interview study in Sweden

**DOI:** 10.1186/s13690-024-01387-1

**Published:** 2024-09-12

**Authors:** Åsa Grauman, Erica Sundell, Jennifer Viberg Johansson, Nina Cavalli-Björkman, Jessica Nihlén Fahlquist, Mariann Hedström

**Affiliations:** 1 Centre for Research Ethics and Bioethics, Uppsala University, Box 564, Uppsala, SE-751 22 Sweden; 2 Department of Immunology, Genetics and Pathology, Uppsala university, Rudbecklaboratoriet, Uppsala, SE-751 85 Sweden; 3 Department of Public Health and Caring Sciences, Uppsala University, Box 564, Uppsala, SE-751 22 Sweden

**Keywords:** Patient education, Breast cancer, Colorectal cancer, Qualitative research, Stigma, Lifestyle

## Abstract

**Background:**

Informing individuals about their risk of cancer can sometimes have negative consequences, such as inflicting unnecessary worry and fostering stigma. This study aims to explore how patients diagnosed with breast or colorectal cancer perceive and experience risk communication, particularly concerning the increased focus on lifestyle behaviors as the cause of cancer.

**Methods:**

Semi-structured interviews were conducted during autumn 2023, with 23 Swedish individuals, aged 34 to 79 years, diagnosed with breast or colorectal cancer. The collected data were analyzed using inductive thematic analysis described by Braun & Clark. The study adopted an experiential orientation grounded in critical realism.

**Results:**

Five themes with ten sub-themes were identified: Thoughts and feelings about the causes of cancer, Moralizing messages and negative encounters, The need to take action, Balancing uncertain risks and a fulfilling life, and Societal benefits of risk communication. The participants expressed that knowledge of the the cause of cancer is closely related to the possibility of taking preventive action against relapses. Ability to take action was also perceived important for their well-being. Therefore, risk information entails both feelings of self-blame and hope for the future. Participants asked for both information and lifestyle support from healthcare professionals. Lifestyle interventions and patient support groups were solicited and perceived as an important aspect of cancer survivals’ well-being, and may help to reduce the cancer-related stigma.

**Conclusion:**

Individuals that have or have had breast or colorectal cancer, including those leading healthy lifestyles, found moralistic risk information offensive, leading to feelings of shame when thinking about other peoples thoughts. Balancing information involves providing transparent, evidence-based information while considering individual and social contexts, avoiding stigmatization and blame, and supplementing information with support.

**Supplementary Information:**

The online version contains supplementary material available at 10.1186/s13690-024-01387-1.

**Table:** 

**Text box 1. Contributions to the literature**
• Patients, with various lifestyles, felt offended by the message of lifestyle-related risk information. They feared it could create preconceived notions about their lifestyle, leading to feelings of shame.
• Patients asked for lifestyle interventions and patient support groups, which they perceived as an important aspect of their well-being.
• Patients suggested more nuanced risk communication, clarifying that breast cancer and colorectal cancer have multifactorial causes, and while lifestyle choices are important, they alone do not fully protect against cancer.
• Patients balanced the potential discomfort of risk awareness against the societal benefits of increased prevention efforts, ultimately supporting the priority of the latter.
• Patients emphasized the need to make cancer a more publicly and openly discussed topic to reduce stigma and instill hope.

## Introduction

Lifestyle plays an essential role in the development of cancer; 44% of all global cancer deaths are linked to modifiable risk factors [1]. Lifestyle behaviors, unlike heredity, are modifiable and managed in everyday life, influenced by habits and values, and closely attached to the individual’s identity [2]. Informing the public about lifestyle-related cancer risk factors enable informed decisions about health. However, beliefs about causation is closely connected to stigma, where the perception of personal control over risk factors increases blame on the patients and the notion that the disease is self-inflicted [3]. Therefore, public information campaigns aiming to raise awareness about the cause of cancer to prevent cancer can potentially inflict stigma on the individual [4, 5]. For instance, to prevent lung cancer, governments in many countries raised awareness of smoking as its primary cause and purposely aimed to change social norms around smoking, in order for it to be perceived as undesirable and unacceptable [6]. This led to the assumption that all lung cancer patients are smokers or former smokers, causing patients to feel blamed for their disease and fear being denied access to care [7]. The fact that smoking became much more common among socially vulnerable groups further contributes to this stigmatization [8]. Stigma is commonly defined as an attribute that differentiates a person or a group from others and links an individual to undesirable stereotypes [9], leading to a loss of status or discrimination [10]. Stigma can be both “enacted,” which refers to the active discrimination of stigmatized people and “felt,” which refers to an individual’s internal experiences and anticipation of discrimination, together with emotions including shame and guilt [9, 11]. Negative consequences of stigma include less allocation for preventive interventions [12], delays in health-seeking behavior, tolerance of discrimination, and mental distress [3, 6]. The stigmatization of cancer patients is also connected to negative perceptions of the disease itself, which patients experience through others’ avoidance or discomfort [7]. These perceptions involve feelings of dread due to perceptions of high mortality, and aggressive, disfiguring and disabling therapies [3, 6, 11, 13, 14].

Previous studies comparing the level of stigma and blame associated with different cancer diseases have shown that lung cancer attracts blame attributions to a higher extent than, e.g., colorectal cancer (CRC), while stigma and blame seem to be attributed to breast cancer (BC) to a very low degree [6, 15, 16]. Hence, the stigma associated with cancer can vary significantly depending on the type of cancer and its perceived causes, reflecting broader societal attitudes and awareness. Female BC is the most common cancer in Sweden, with 11,327 cases in 2021 [17]. Colorectal cancer is the fourth most common cancer in Sweden, with 7,583 cases in 2021 [17]. Both BC and CRC have multifactorial etiologies including heredity, lifestyle factors and environmental factors [18]. Lifestyle related risk factors were linked to 15% of all BC cases in Sweden and 30–36% of all CRC cases in 2018 [19]. Common lifestyle related risk factors for BC and CRC are high alcohol intake and obesity, while physical activity is a common protective factor [18]. High exposure to female hormones is an additional risk factor for BC, as well as high breast density [18]. Risk factors for CRC also include a high intake of processed or red meat, smoking [20], and inflammatory bowel diseases [21]. About 41% of the adult Swedish population have a risk consumption of alcohol [22] and about 50% are overweight or obese [23].

In several countries, including Sweden, the awareness of smoking as a risk factor for cancer is high [24, 25]. However, the awareness is low for other risk factors including alcohol intake and low fruit and vegetable consumption for cancer in general [24, 25] and for BC and CRC specifically [26–28]. Awareness of cancer risk factors also vary both between countries and within countries due to socioeconomic differences [26, 28, 29].

As awareness of factors such as obesity and alcohol as causes of BC and CRC increases, it is possible that the blame attributed to patients may also rise, as has been observed in the past with smoking as a cause of lung cancer. To ensure that future risk communication about lifestyle and cancer is beneficial while minimizing harm, it is crucial to understand the perspective of patients, since stigma includes thoughts and feelings of the individual and not merely actual treatment from society. Since BC, CRC, and unhealthy lifestyle habits are common in Sweden, this is a prioritized research area. This study aimed to explore how patients with BC and CRC perceive and experience lifestyle related risk communication, and the increased focus on lifestyle behaviors as the cause of BC and CRC.

## Methods

The study was a qualitative semi-structured interview study. The consolidated criteria for reporting qualitative research (COREQ) [30] have been followed to ensure transparent and quality reporting (Additional file 1).

### Recruitment & study population

The participants were recruited with the help of two national patient associations (one representing BC and the other representing bowel diseases). They sent information about the study to their members via email. Participants were offered a gift certificate worth 150SEK (≈ 13 €) as a token of appreciation. Inclusion criteria included being aged 18 or above, having or had BC or CRC, and being able to read and speak Swedish. Individuals willing to participate contacted ÅG. The interest to participate among individuals with BC was higher than the need for participants; hence, a purposeful selection was made with the aim of recruiting individuals of various ages from different parts of Sweden. As all individuals with CRC who expressed interest were recruited for participation, *convenient sampling* was used for this subgroup. The final sample size was guided by the concept of information power, which is suitable for exploratory studies that do not apply grounded theory [31]. The study had a broad aim, and the participants had diverse experiences and perceptions of risk communication, necessitating a larger sample size for cross-case analysis. However, the high-quality dialogue in most cases and the study’s grounding in theories about stigma reduced the number of participants needed [31]. Participants’ characteristics are presented in Table 1.

**Table 1:** Participants’ characteristics

	*N*	Median, range
Total	23	
Cancer diagnosis		
*Breast cancer*	11	
*Colorectal cancer*	12	
Time since diagnosis		4 years, range: 8 months– 15 years
Sex		
*Female*	17	
*Male*	6	
Age (years)		58 year, range: 34–79
Born in Sweden		
*Yes*	21	
*No*	2	
Educational level*		
*Primary school*	1	
*Secondary school*	4	
*University*,* post-secondary education*	17	
Training as a health professional		
*No*	20	
*Yes*	3 (1 physician, 1 registered nurse, 1 nurse assistant)	

*1 = missing

### Data collection

Participants were given the choice to participate in an interview face-to-face, online (video call through Zoom), or by telephone. This flexibility aimed to make them as comfortable as possible and to overcome the barrier of geographical distance to increase participation, although at the expense of losing some control over the interview environment. The approach worked well and the participants appeared to feel at ease. However, due to poor internet connections, we had to switch from Zoom to telephone calls for a few participants, which had the downside of not being able to see each other. The participants could also choose if they wanted to have the interview individually or take part in a focus group discussion (FGD). Decision to combine individual interviews with FGD was made both for pragmatic reasons and to enhance understanding of the topic through gaining multiple perspectives [32]. The preferred option among the participants was individual interviews via Zoom. Although several group interviews were scheduled, logistical challenges arose as many participants needed to re-schedule their appointments. The interviews were conducted in Swedish by ÅG (PhD, PI) and ES (RN, MSc), both females, during the autumn of 2023, using a semi-structured interview guide that had been pilot-tested before the interviews. ÅG had previous experience with qualitative studies, while ES, a research assistant, had experience in patient communication. The interview guide covered the following areas: causes and risk factors, experiences with risk information, reactions to risk information, benefits and cons of risk information and preferences for risk information. Examples of probing questions were: “how did that make you feel?”, ”can you tell me more about that?”, and “what do you think about that?” The FGD interview guide covered the same themes. However, the participants were encouraged to speak freely and to address each other directly, and probing questions e.g. “do the rest of you agree?” and “could you tell us more about that?” were asked to facilitate a discussion. The participants were also asked background questions at the beginning of each interview (Table 1). The researchers had no prior relationship with the participants before the interviews. One interview was conducted face-to-face, three over the telephone, and 17 via Zoom, including the one group interview with three participants. The individual interviews ranged from 19 to 58 min (median 30 min). The FGD lasted 72 min. The interviews were audio-taped and transcribed verbatim (divided by ÅG and ES, as well as by a professional transcription company). All personal identifiers were removed from the transcripts.

### Data analysis

The data were analyzed inductively using thematic analysis according to Braun and Clark [33, 34], chosen for its flexible and structured approach that provides a rich and nuanced account of the data. The researchers applied an experiential approach grounded in critical realism that entails exploring subjective experiences, recognizing an independent reality while acknowledging that both participant experiences and researcher interpretations are shaped by cognition, language, and social contexts. Thematic analysis recognizes the researchers’ subjectivity as a valuable resource in the analysis process. Themes are seen as a result of the researchers’ active choices, rather than something that “emerges” from the data [34]. The analysis started with familiarizing with the data by repeatedly and actively reading the transcripts. Five female members of the research group each read and coded the same three interviews individually and then met to code one of the interviews together, aiming to enhance understanding rather than reaching consensus. ÅG and ES coded an additional interview jointly. The coding and reflections on the other two interviews were handed over to ÅG to incorporate into the remaining coding. The remaining interviews were coded by ÅG. The group reconvened when all text was coded to initiate the sorting of codes into potential themes. In the next step, ÅG reviewed the themes while continuously going back and forth between transcripts, codes, and themes. During this process, themes were either collapsed or broken down. In a joint discussion, the research group defined and named the final set of themes and sub-themes [33]. The codes from the focus group discussion were combined with those from the individual interviews, as they largely overlapped. The software Atlas.ti 9 was used to organize the codes and initial themes. Examples of codes, quotations and themes are presented in Additional file 2. The research team comprised individuals with backgrounds in public health, nursing, medicine, and ethics, with extensive experience in qualitative research and knowledge of risk perception and stigma. The researchers openly and critically discussed their interpretations along the analysis process to enhance reflexivity.

## Results

Five themes with ten sub-themes were identified (Table 2). Although some participants did not perceive any negative aspects of risk communication, the results mainly highlight raised problems and solutions and describe contradictions and ambiguities related to the topic.

**Table 2:** Overview of the results

Themes	Sub-themes
1 Thoughts and feelings about the causes of cancer	1.1 Scrutiny of one’s lifestyle behaviors and exposures
	1.2 The possibility of influencing the cause entails both a sense of hope and guilt
2 Moralizing messages and negative encounters	2.1 The negative impact of moralizing messages
	2.2 Negative experiences of comments and encounters
3 The need to take action	3.1 Desire to prevent cancer relapse and enhance well-being
	3.2 Soliciting healthcare professionals to inform and support
4 Balancing uncertain risks and a fulfilling life	4.1 Room for action and agreement with societal norms
	4.2 Personal way of life is not easily sacrificed amidst uncertain risks
5 Societal benefits of risk communication	5.1 Increase knowledge to prevent cancer in society
	5.2 Reduce stigma and provide hope

## Thoughts and feelings about the causes of cancer

The participants often found themselves asking, “Why me?” after being diagnosed with cancer, and expressed that risk communication could help provide understandable answers to this essential question.

### Scrutiny of one’s lifestyle behaviors and exposures

The participants contemplated why they had developed cancer and whether they could have done something differently to prevent the disease, especially shortly after the diagnosis.

“The first question is, will I die? And the second question is, why did I get it? So it would have been good to have someone better, someone to talk to, who had time to talk about it and explain.” (Participant 8, female, age range 40–49, BC).

This prompted self-reflection on their lifestyle leading up to diagnosis, including not only behavior but also encompassing general life factors, such as stressful work. Participants extensively sought answers for the cause of their cancer in different sources, including internet searches, lectures, and in consultations with physicians. Common physician responses included coincidence, bad luck, or unknown causes, leaving some participants dissatisfied and desiring further discussion.

Participants often compared their own lifestyle with those around them, and with people on social media, as a reference point for what is normal or in terms of doing better or worse than others. Many reasoned in terms of doing right or wrong. Many considered themselves living healthy yet still developing the disease, evoking strong feelings of injustice and incomprehension. Others found comfort in knowing they had lived a healthy life, or felt acceptance and confidence in having made conscious choices to take the risks associated with unhealthy habits.

“…I’ve been to lectures, and really tried to figure it out… I’ve been training and living, I think, fairly healthy… it somehow felt like a punishment, but then you go crazy, you google like crazy; yes, I’ve asked a lot, but no, you don’t know why you got it really; it was probably fate and bad luck and whatever it might be. I think that my illness was unfair to me. I think based on how I had lived before, and then I blame myself a little; if I had done something else or lived another way… but I don’t know what. Would I have gotten the cancer then?” (Participant 11, male, age range 60–69, CRC, FGD).

Most participants were uncertain about the cause of their cancer. Some considered hereditary factors but had negative result from genetic testing. Many attributed their own disease to stress, sometimes as a rational conclusion after excluding other possibilities. Pre-existing knowledge of cancer and risk factors varied; some were well-informed, while others were unaware of the link between lifestyle and cancer, which affected their understanding of causation.

### The possibility of influencing the cause entails both a sense of hope and guilt

Reflecting on whether they could influence the cause generated ambivalence among participants. Knowing that the cause is unchangeable provided some participants with reassurance that they had done nothing wrong. Conversely, some perceived hereditary causes negatively since they could have consequences for their children.

“I didn’t have the genetic marker. I know I cried and laughed, alternating, and wrote to my children and said, ‘here you get the best present’. It would have been really tough to live with, I think. If I had passed it on. Of course, I would have done it completely unknowingly, but it would still feel that way.” (Participant 16, female, age range 70–79, CRC).

Participants expressed that modifiable lifestyle causes present an opportunity to prevent relapses, and a possibility for their children to prevent cancer. However, the notion of influencing the cause induced self-blame, which was perceived as heavy to carry in their already tough situation.

“When you are told that you have been unlucky, it is in a way ‘Okay, I haven’t done anything’, but somehow you still feel that you want to be able to do something.” (Participant 22, female, age range 50–59, BC).

## Moralizing messages and negative encounters

This theme reflects how the message of personal responsibility in risk communication, and negative experiences of interaction with healthcare professionals can adversely affect participants.

### The negative impact of moralizing messages

Participants found the rhetoric of risk communication judgmental and moralizing, implying that a healthy lifestyle is a shield against cancer, indirectly assigning blame to the individual for their illness. While participants usually did not personally blame themselves, they felt offended by the message, fearing it could create preconceived notions about their lifestyle. Many believed that the oversimplified message also raised false hopes, suggesting that a healthy lifestyle alone safeguards against cancer.

“I can think that it would be a little unfair if a person who has not had cancer reads such an article, then one might think that ‘yes, but I have lived the right way because I have not had cancer’. Although other people who have lived exactly the same way might get cancer. Sometimes, there is an image that if you are physically active and eat a certain diet, you won’t get cancer. So, then if you get it, then you have done something wrong.” (Participant 17, female, age range 40–49, BC).

Some participants acknowledged that they needed to exercise more, eat healthier, or lose weight, but struggled to do so on their own. Hearing about risk information triggered feelings of guilt and worry for them. Some feared that the stress caused by this information might lead to increased eating or drinking as a coping mechanism.

To alleviate the blame ascribed to patients, they suggested more nuanced risk communication, clarifying that BC and CRC have multifactorial causes, and while lifestyle choices are important, they alone do not fully protect against cancer. Comparing to smoking and lung cancer, where patients are more likely judged, they underlined that CRC and BC lack a single key risk factor, which could reduce the blame directed at patients. Some emphasized the importance of discussing broader societal influences on individual habits, acknowledging factors such as childhood circumstances and societal changes that contribute to unhealthy habits. One woman reflected on the underlying values of risk communication, perceiving society as individualistic, where individuals bear sole responsibility for their life choices.

“I have also noticed that this sense of guilt often inhibits people from taking action or from choosing another way that could lead to a better life for them. It becomes yet another expectation they must meet. I notice that I have been greatly affected by this increased communication of, you are responsible for making yourself sick or healthy. I find that unsettling, even today, … ‘yes, yes, but she’s so fat because she doesn’t exercise’ or whatever it is; it’s always the individual’s fault. But there are many dimensions to why people are the way they are. And this aspect is missing in health communication.” (Participant 6, female, age range 60–69, CRC).

### Negative experiences of comments and encounters

Participants shared experiences where people they met commented on the causes of their cancer or their lifestyle choices. Some were puzzled when a seemingly healthy person was diagnosed with cancer. Certain participants felt that the questions reflected people’s attempts to assess their own risk. Others perceived the questions about the cause as negative scrutiny. One woman felt shame, thinking about what other people thought of her lifestyle despite living healthily. However, many of the participants did not feel that others blamed them for their cancer.

“I have been asked, ‘Why did you get it?’ What have you been eating? What have you been doing?’ It makes me both sad and angry. It feels like I’m being shamed, like I have done something wrong because I got cancer. People question why I got it, when even I don’t know myself. It is lifestyle habits that they are talking about. And I don’t think I could have lived any better. I don’t drink, I don’t smoke. I’ve been exercising my whole life. Yet, I feel ashamed that I still got cancer. I think other people must wonder about me, like how does she live? Yes, but I haven’t done things like drinking Coca-Cola every day and destroying my intestines… Still, I feel this sense of shame.” (Participant 15, female, age range 50–59, CRC).

Two participants with BC were told by others that their consumption of diet soda had caused their cancer, although one participant’s physician helped dismiss this notion. One woman thought CRC patients with stereotypical features might be judged more easily, such as an obese man who consumes barbecued red meat and alcohol. Another overweight participant had a similar reflection, noting that it is easier to blame obese individuals because their weight is clearly visible.

Participants who were overweight shared negative encounters with healthcare professionals, where their weight was frequently raised as a health concern, yet no support was offered for weight loss. Feeling blamed for their weight was described as burdensome, adding to the challenges of cancer treatment. One woman felt that mistreatment of overweight patients was socially acceptable, attributing this to prevailing societal norms.

“One becomes sad. One becomes angry… I don’t want to be overweight. I don’t feel good about it… then I get more weight on me that, like… my mood gets worse mentally because I constantly feel… that knot in my throat. Like, hey, you’re overweight. Yeah, but, I’m not stupid, I know. And this sense of guilt that you get, it’s difficult to bear.” (Participant 2, female, age range 50–59, BC).

“When offering lifestyle advice, it is important not to place blame because, as a cancer patient, you’re already so profoundly affected by your disease, which scares you so incredibly much; so, you have to be kind and considerate because, like everyone else, you can’t do everything, and certain circumstances make you unable to…” (Participant 8, female, age range 40–49, BC).

Many participants emphasized the importance of the quality of communication rather than just the content. They believed that by conveying empathy, warmth, and understanding, would reduce the feeling of being blamed.

“That one should be humble and understanding. That’s probably the most important thing. […] It’s important with body language… That is, it’s about sensing what works for both individuals, and to sort of dare to place a hand on a shoulder or, yes, and get a feel for if it even works with a hug, so sometimes all you need is a hug.” (Participant 3, female, age range 30–39, BC).

## The need to take action

This theme reflects participants’ desire to take action and their request for support from healthcare professionals to do so.

### Desire to prevent cancer relapse and enhance well-being

Many participants felt a strong need to take action post-diagnosis in order to improve their situation, driven by the fear of cancer relapse. Some actively sought ways to regain control and were willing to try many different methods. Specific diets, such as incorporating blueberries or beetroot juice, were commonly adopted, reflecting widespread discussions among patients about foods believed to be beneficial for cancer prevention and treatment.

“What you can do yourself to prevent it, it is a way to gain control over the situation; you have ended up in a situation that you absolutely did not ask for. It has caused a, it’s a big trauma, you don’t know how it will end, and getting control, really, that’s what it’s all about. You can’t make that much of a difference, but if you can influence something, it’s how you live; it’s about control.” (Participant 8, female, age range 40–49, BC).

“I want to believe that you can make a difference yourself. Even if you think that I have a risk of relapse, but I can’t do anything about it, well, you kind of want to think that I can do something. I can do something myself for my own health.” (Participant 4, female, age range 60–69, CRC).

Some valued healthy lifestyles for general well-being, not just for disease prevention. Others considered information about lifestyle choices as more relevant post-diagnosis, as they had previously never considered themselves vulnerable to cancer.

### Soliciting healthcare professionals to inform and support

Many participants sought information on ways to influence their situation, but felt overwhelmed by the sheer amount of information available. They struggled to navigate it alone and turned to health professionals for advice but were often disappointed when told to simply maintain their current lifestyle. Some participants felt disregarded, believing that healthcare professionals avoided the question, possibly to avoid blaming patients. A few participants were satisfied with the information they received.

Participants thought that the healthcare professionals should offer lifestyle guidance, considering their expertise and credibility. However, they described timing as crucial, especially since many patients are in a state of shock post-diagnosis. Some felt unsupported and wished for access to dietitians, physiotherapists, or lifestyle coaches. One noted the costly nature of support outside the healthcare system, creating inequality. Many participants solicited psychosocial support to help manage emotional stress, suggesting that patient support groups could be a solution for both psychosocial and lifestyle support.

“Healthcare today is not so damn good. They do what they have to. They remove the cancer; you get chemotherapy. Cheers, bye. You don’t get any… I just asked about what to eat afterwards, if I should think about anything or things like that. You can eat exactly what you want, bye. They don’t have time for things like this…, and then you just say goodbye.” (Participant 15, female, age range 50–59, CRC).

“That you find personal responsibility without assigning blame. And that you make it exciting, positive… Or set up self-help groups. They can lead each other; they listen to each other; talk and listen… it doesn’t have to cost a lot.” (Participant 10, female, age range 50–59, BC).

## Balancing uncertain risks and a fulfilling life

In contrast to prior themes, some participants hesitate to comply with risk communication due to its perceived weaknesses, and instead weigh the perceived benefits against each other.

### Room for action and conforming to societal expectations

Participants viewed constant warning-based risk communication negatively, finding recommendations difficult to relate to and too detached from daily life. They saw warnings as limiting and believed lifestyle advice should emphasize achievable goals and promote small, positive changes. Recommendations should align with societal norms and culture and be realistic.

“Today, there are a lot of warnings for almost everything. And then it becomes difficult to relate to them, and you just tune them out. After all, I still need to eat… but all you hear, it becomes like, you shouldn’t do this, and you shouldn’t do that. In the end, yes, what should I do then? […]Based on the National Board of Health and Welfare’s view on alcohol habits, it seems that I have been an alcoholic for a long time. Given that, you might as well continue. It kind of becomes so unattainable and somehow… then it doesn’t feel like you’re doing anything wrong, even though the research probably says you are… although that’s not the general perception of normal alcohol behavior.” (Participant 9, male, age range 50–59, CRC).

### Personal way of life is not easily sacrificed amidst uncertain risks

Some participants expressed a desire to maintain their pre-cancer lifestyle. For example, by enjoying food and beer with loved ones and not deviating from their social context, believing it added value to their lives. One woman, who has teenage children, tried to avoid instilling negative attitudes toward food.

“I think I should be allowed to live a reasonably normal life. Yes, but with my children, I have always encouraged food as something social, where everyone eats the same meal together. Not that it should be a big deal, and everyone has their own little diets.” (Participant 17, female, age range 40–49, BC).

“I do drink alcohol, but I also think there’s a balance to strike in life. It’s about enjoying life, not to stress, to stay healthy, to sort of not live like an ascetic, because I don’t think that feels good either. It’s about the big picture… enjoying life is also important for overall well-being.” (Participant 12, female, age range 50–59, CRC, FGD).

Some reasoned in compensatory terms: with an overall healthy lifestyle, they felt entitled to indulge in some areas. Others dismissed specific risk factors, noting instances where unhealthy individuals remained cancer-free, or very healthy individuals still developed cancer. Meanwhile, many participants perceived the risk reduction from lifestyle changes as uncertain or referred to the lack of scientific evidence on risk factors. To aid patients in balancing risk reduction and lifestyle changes, they requested more specific risk information, including precise percentages of risk reduction and guidance on safe consumption levels of sweets. Many sought personalized risk information tailored to their circumstances, ideally communicated during health check-ups or screening visits. Overall, risk information was deemed too vague.

“One could have a conversation about prevention… now we will talk about you and your conditions. What kind of lifestyle habits do we have here and what kind of medical history do you have. What can we see in the family’s genetics and so on. And then we go through it in a fact-based… someone who sort of takes an interest in me and, based on my conditions, tries to get an overall picture. But when it just becomes fragmented, warnings about this and now we warn about this.” (Participant 9, male, age range 50–59, CRC).

“If you could say, what is the most important of them? Is it the genes or is it what you eat and drink or is it stress or other lifestyle habits? So yes, if you are told that the genes mean 50% maybe and the others then mean fifteen or twenty, something like that.” (Participant 5, male, age range 70–79, CRC).

## Societal benefits of risk communication

This theme describes participants’ view on the societal benefits of communicating risks to the public.

### Increase knowledge to prevent cancer in society

Many participants emphasized the importance of informing the public about the link between lifestyle and cancer to prevent future cases. Some accepted potential downsides, such as blame or worry but believed that if it meant that cancer could be prevented, it was worthwhile.

“And if it then becomes offensive or condescending to someone, then so be it. This should go out to everyone. I think that if we can prevent illness and death by informing, then it is good. There we have the recipient who has to receive it. And if we then start talking about it, maybe it will become more open. And if we don’t talk about it, nothing will happen.” (Participant 7, female, age range 50–59, BC).

Many participants noted low public awareness of the link between lifestyle and cancer, especially regarding the risks associated with alcohol consumption. They believed the public should be informed to make better choices. Increased awareness was seen as enabling preventive efforts in schools and workplaces, thereby fostering a healthier society.

“I think it’s fantastic because this could mean that maybe employers and others have to take a different responsibility and… I think it should be raised much, much more and early. At the preschools, let them be outside to play and move around, fresh air, good food.” (Participant 21, female, age range 40–49, BC).

### Reduce stigma and provide hope

Many participants believed that effective risk communication could reduce the stigma surrounding cancer by encouraging people to talk about it openly. They stressed the importance of reducing fear associated with cancer and instilling hope by highlighting that there is a lot that you can do, such as lifestyle modifications, early symptom recognition, and participation in screening programs. Additionally, they advocated for informing the public about improved treatment outcomes and the possibility of leading a fulfilling life post-treatment.

Participants with CRC highlighted the taboo surrounding the disease, particularly due to its association with bowel health. One participant referred to CRC as “ugly cancer,” noting its lower status and limited media attention compared to other cancers like BC. They emphasized the need for increased public awareness and information campaigns similar to those conducted for BC.

“And it’s not something people want to hear; it’s not something you bring up like that when you’re out at a party… We go about our lives and… it’s somehow assumed that you’re healthy… like others. And if you deviate from that… especially with an illness, you don’t really want to know. And it probably depends just as much on fear, because it could happen to you next… I myself have had chronic diarrhea from this. And it becomes a stigma for me. And it’s not something you might really go and explain to the average person… well, there, you wish that our society might have come further in some respects.” (Participant 9, male, age range 50–59, CRC).

*“I think screening could open up the dialogue…*,* yes*,* imagine if granddad is at the screening*,* then*,* we could talk about such things as diet*,* how important that part is too… it would have made the discussion easier. It would have been much easier to talk about it.*

*- I also think we need to make it a little less taboo; nobody talks about it […] The word cancer sounds… I think many people associate it with death and if it can be removed somehow.*
*-And to explain… perhaps information about what cancer is*,* how it develops*,* de-dramatize based on the fact that you can influence the situation*,* but also that it is a dangerous disease. This form of cancer*,* when you get your diagnosis*,* it is far from the end.” (Group discussion*,* one female*,* two males*,* age range 50–60*,* CRC)*.

## Discussion

Overall, all participants agreed that providing information about lifestyle risk factors was positive and needed. However, they also identified many negative aspects of the information and gave suggestions for improvement.

This study demonstrates how risk information intertwines with individuals’ existing knowledge, experiences, and to norms of society. Patients receive risk information from various sources, including healthcare professionals, public health authorities, social media, and news outlets. Participants felt a need to discuss and interpret this information and sought assistance in determining its relevance. Healthcare professionals’ communication about lifestyle and obesity is likely to evoke strong reactions influenced by patients’ past experiences, potentially resulting in stronger and more negative responses than expected solely based on objective information. Participants in our study raised several unmet information needs, which is a recurring theme in previous research conducted in US [3], Mexico [35], and China [36, 37]. For example, information needs and psychosocial support have been found to dominate the unmet needs among Italian patients with BC [38]. The lack of guidance from healthcare professionals can be problematic, as patients are left to seek information on their own, for example, on social media, leaving them exposed to incorrect information that could, in the worst-case scenario, have harmful consequences to their health [3, 35–37]. Meanwhile, the participants perceived it as the healthcare system’s responsibility to educate them. The issue appears to be an essential question for patients’ peace of mind. When patients asked their physician about the cause of their cancer, they often got the reply that it was bad luck, coincidence, or that it was unknown. Participants felt disappointed with these responses, wanting a further discussion. Some participants even felt that the physician was withholding information to avoid causing harm, which, as other studies have shown, can evoke feelings of anger [3] and have a negative impact on the doctor-patient relationship, resulting in decreased trust.

As risk numbers are calculated at an aggregated level, it is not possible to define the cause behind one individual’s disease, especially when it has a multifactorial etiology. This discrepancy between group-level risk and individuals’ experiences poses a challenge in risk communication, not only in determining the cause but also in communicating about preventive actions [39, 40]. This study suggests that patients’ experiences could be improved through more transparent and empathetic discussions with healthcare professionals. Also, a way to support patients could be to re-direct the focus from identifying the cause of cancer to emphasizing the possibility to take action, which participants identified as an important aspect for their well-being.

Making lifestyle changes was related to the participants’ need to take action and was described as a way for them to regain a sense of psychological control. Previous studies have also found that both BC and CRC patients often make dietary changes as a way to cope with the disease [41], including managing stress and the fear of cancer recurrence [37, 41]. Lifestyle interventions can, therefore, be considered as part of psychosocial care and have been shown to help patients maintain a positive self-identity [42] and reconnect with their normal lives [37, 41, 43].

Participants perceived difficulties in managing their lifestyle on their own and noted a lack of support from the health care system. Similar experiences were reported among patients with colon cancer in Mexico, who emphasized the need for support groups focusing on emotional support, information, and empowering cancer patients, similar to initiatives currently offered by civil society organizations. They, like the participants in our study, experienced that much of the existing activities in society were intended for women with BC [35].

Participants in our study who were unaware of lifestyle factors as cancer risk factors did not exhibit self-blame. A previous study found that CRC patients who attributed their condition to uncontrollable causes had lower levels of anxiety than those attributing it to controllable causes [44]. Increased awareness of the link between lifestyle and cancer, however, may increase the attributed blame on patients, thus impacting their psychological well-being. Nevertheless, patients who attribute their cancer to controllable causes or are facing mental distress may show a greater readiness for and benefit from lifestyle interventions.

Personal control over the causes of cancer is closely related to the stigma associated with the disease. In our study, participants were offended by lifestyle-related information that they perceived placed blame on patients and created pre-conceived notions about their lifestyles. Unexpectedly, also very healthy individuals were offended by such information, as they felt ashamed and feared what others might think of their lifestyle choices. The participants appeared to perceive significant discomfort due to the discrepancy between the unhealthy stereotype associated with cancer and their personal identity as healthy and active individuals, fearing they would be viewed differently by others. The participants suggested that the blame placed on patients could be reduced by emphasizing the multifactorial and societal influences on lifestyle behaviors.

Interventions aimed at reducing health-related stigma can include both individual- and structural level approaches [45, 46]. Internalized (“felt”) stigma can be reduced through therapeutic interventions or support groups [45–48]. The participants in this study also suggested support groups as a way of reducing blame on patients, as well as communicating with empathy, warmth, and understanding the individual’s perspective. Effective strategies for addressing socially enacted stigma at a structural level include communicating positive stories of stigmatized groups, implementing contact-based training and education programs for health professionals [47]. Communicating in a positive way was something that was also highlighted by participants who believed that stigma could be reduced by raising awareness about cancer in a positive and hopeful manner.

### Strengths and limitations

The credibility influences the trustworthiness of qualitative research and was strengthened in this study by the involvement of multiple researchers in the investigation and analysis. The researchers’ knowledge of qualitative methodology, along with their complementary theoretical knowledge and diverse clinical experience, also enhanced the study’s credibility [49]. This study also combined individual interviews and FGD, however, only one small FGD is not enough to achieve qualitative triangulation. Conducting several FGD had strengthened the study, since FGD can complement individual interviews by adding a more broad contextual perspective [32].

Transferability also influence the trustworthiness. It is possible to assess transferability to other settings with a thorough description of the study sample [50]. Our results, to a high extent, correspond to study results from both China [36, 37] and Mexico [35], indicating that although our study was conducted in Sweden, the findings indeed have common themes described across varying cultural contexts worldwide. In order to attain a diverse and heterogeneous sample we recruited individuals with varying viewpoints [50]. We anticipated that patients’ views could differ with age, sex, time since diagnosis, educational level, medical or caregiver training, and assessed these variables. During the interviews, it became evident that the participants’ pre-understanding about the link between lifestyle and cancer varied from none to being highly informed. The majority of the participants were highly educated and born in Sweden; therefore, the results may not reflect the views of other groups in society, e.g., immigrants. We did not assess actual lifestyle or body weight, but the participants brought it up themselves, and it appeared that most of them perceived their lifestyle as quite or very healthy. It is possible that individuals who perceive themselves to have unhealthy behaviors were less willing to participate in the study due to perceived blame. However, two participants brought up their body weight and how it related to previous experiences with the healthcare system and how they reacted to risk information. The study sample varied greatly in all these perspectives, which is a major strength of this study. We refer to patients in this study, but it is important to note that we recruited both cancer patients and cancer survivors who are now declared cancer-free. However, we perceive this as a strength since the time perspective and disease stage offer further variety in perspectives. We do not believe the gift cards influenced study participation, as the amount was low (barely covering a movie ticket) and several participants expressed the study’s importance.

## Conclusion

Risk information is not transmitted or received in isolation. Scientific findings are contextualized through the receivers’ experiences and societal norms. The dissemination of risk information can also influence changes in societal values and attitudes, particularly regarding issues of responsibility, personal accountability, and societal perceptions of specific lifestyle behaviors and body weight.

Risk communicators must consider societal values and public perceptions, designing messages that are respectful, inclusive, and mindful of the diversity within the audience. For cancer patients, risk information pertains to both the causes and the potential for preventive measures. Lifestyle interventions are also linked to psychosocial well-being and are necessary for patients to effectively utilize this information.

Patients, even those with very healthy lifestyles, may feel offended by moralistic risk information. Striking a balance involves: (a) providing transparent, accurate, evidence-based information, while (b) considering the individual and social contexts, (c) avoiding stigmatization and blame, (d) and supplementing information with support.

## Electronic supplementary material

Below is the link to the electronic supplementary material.

Supplementary Material 1

Supplementary Material 2

## Data Availability

The data that support the findings of this study are not openly available due to reasons of sensitivity and are available from the corresponding author upon reasonable request. Data are located in controlled access data storage at Uppsala university.
