# Supplementary material for: Perceptions of lifestyle-related risk communication in patients with breast and colorectal cancer: a qualitative interview study in Sweden
Source: Arch Public Health. 2024 Sep 12;82:154. doi: 10.1186/s13690-024-01387-1 (PMC11391682; doi:10.1186/s13690-024-01387-1)
Supplement: Supplementary file 2 — Supplementary Material 2 [file 13690_2024_1387_MOESM2_ESM.pdf]

Additional file 2. Example of analysis process

Please keep in mind that the interviews were in Swedish and that the text in this document has not been professionally translated and may not be 100% correct.

Themes	Sub-themes	Codes (few examples)	Quotes (few examples)
1 Thoughts and feelings about the causes of cancer	1.1 Scrutiny of one's lifestyle behaviors and exposures	Why me? The first question that comes to mind.<br>Want to know the cause of cancer.<br>Seeks explanation for cause.<br>Reason important to understand.<br>Did everything right but still sick.<br>Feeling of injustice, done everything right.<br>Sick despite healthy habits, incomprehensible.	Is there something, is there something… what have I done wrong do you think or what, yes…
			Because one of the very first questions you ask yourself is, why me? Could I have done something differently?
			For others it might not be, for me it is because I am... I am such a cause and effect person. No, for me as a person it is important to know why, why I am affected by this. So that's why I searched and found out. So yes, for me it is.
	1.2 The possibility of influencing the cause entails both a sense of hope and guilt	Double feelings about modifiable causes.<br>Heavy and good at the same time.<br>Positive if the cause can be influenced yourself.	So that it also arouses difficult feelings but it can also be positive, so that it is both...
			It's probably the case that you would prefer to be told that it's not really influenceable, because then I haven't done anything anyway.
			Ability to influence the risk is good. Then I can think about it in the future or for future children or things like that. But myself, well... I've already had it. But for the future, it's probably good if there is, as it were.
2 Moralizing messages and negative encounters	2.1 The negative impact of moralizing messages	Messages about lifestyle give an unfair picture, state patients' habits as bad.<br>Wrong to single out breast cancer among other diseases<br>Risk information makes one feel that one should have done more	So what I might think is a bit unfair is, yes, but if a person who has not had cancer reads such an article, then you might think that "yes, but I have lived the right way because I have not had cancer". Although other people who have lived exactly the same way might get cancer. So, yes.
			yes, but if it's the same for different diseases then you don't need to point out which diseases I think. Yes, but because it will be... that is, if you have, yes, but if you sort of inform a lot about "this is how you should live in order not to get breast cancer" and

			you get it anyway. Does it also in some way imply guilt that you, yes, you have not lived in the right way.
	2.2 Negative experiences of comments and encounters	Health professionals speaks in a mean tone that is guilt-inducing.<br>No experience of judging others.<br>Receives bad treatment from health professionals because of weight<br>Weight is stigmatized because it is visible	And that there is a nuance that it is sort of socially accepted to have this slightly mean tone in the way you talk about obesity and overweight. And a tendency towards debt settlement.
			But I think you... feel like you got what I was trying to say anyway. That it can be very tough to take the blame for being overweight or something like that after a difficult and, for many, very tough treatment. Because you get that from society in general as well. That, you're big and stuff. It can be quite tough.
3 The need to take action	3.1 Desire to prevent cancer relapse and enhance well-being	Trying to influence what is possible.<br>Thought a lot about lifestyle<br>Very responsive to advice after diagnosis<br>Want to know how relapse can be prevented<br>Movement is important to feel good.	Precisely this feeling of wanting to be able to prevent relapse as well
			Yes, but it's great to know what to avoid above all, I think that's the best thing about what you put into yourself. But, then I like and I said, like this that it pushes you to be outside and move and... or exercise, call it what you want, it's movement for the body, that it's important. And I think it's good information simply but I sometimes think it's too cheesy the information
	3.2 Soliciting healthcare professionals to inform and support	Requests support with lifestyle.<br>Lacking support from healthcare.<br>Would like a supportive coach.<br>Support must be bought by individual, expensive and unfair.<br>Self-help groups are missing.	And maybe a little more support, like around... It was, so the treatment itself and stuff like that and when you were in there was good, but there was like no support around. This thing with training and that you had to take it on yourself. And that, it went pretty well for me, but maybe not everyone can handle it.
			Like a coach who could kind of follow you for a longer period of time and help and kind of follow up on you a little. Help to push a little, to be able to hold on. So, I have so much to do and think about and deal with, that I can't seem

			to manage everything by myself, but you need someone who could help support a little.
			There is also a lot to do there, just put people in self-help groups and talk to each other and help each other. It costs nothing. You don't have to pay a healer several thousand to get it, but you can do it just by being a group of eight who sit and listen to each other and support each other. There are simple solutions that the healthcare system does not use.
4 Balancing uncertain risks and a fulfilling life	4.1 Room for action and agreement with societal norms	Everything is dangerous, there is no room for action.<br>Too many warnings turn people off.<br>Advocates lower requirements, small changes	Yes, if you were to always list what is dangerous then according to the National Board of Health and Welfare and things like this, or whoever it is who says that everything is dangerous, then there is not much left. Then there really isn't much left, so then it becomes negative.
			It has to feel relevant and it has to feel achievable. We have just recently heard here the National Board of Health and Welfare's view on alcohol habits, for example, and then you can state that you have been an alcoholic for a long time. Based on that, you might as well continue. It seems to be so unattainable and in some way... which most people feel is so wrong, so then it doesn't feel like you're doing wrong, although the research probably says that you... it's not the general perception of how a normal alcohol behavior is perhaps. And then you get... I have a twenty-year-old daughter myself and I see completely different alcohol habits to relate to... the relationship with... so you could say, based on my age category in relation to alcohol.
	4.2 Personal way of life is not easily sacrificed amidst uncertain risks	Takes risks based on conscious choices.<br>Weighing the risk of alcohol/unhealthy food against the pleasure<br>If you like meat you will eat it.	But since there are lifestyle factors, you kind of have to... then you have to relate to your own lifestyle and be responsible for the risks you take, so to speak. And that's how it is in everything you do.

			I choose to go towards red man sometimes when I see that there are no cars around, it is clear that I expose myself to an increased risk
			I also drink alcohol and so, then I think that there is also something in the other scale which is to enjoy life, not to stress, to stay thin, to sort of not live a life like an ascetic, because I don't think so rather you feel good, so that's the whole thing there,
5 Societal benefits of risk communication	5.1 Increase knowledge to prevent cancer in society	Information is positive despite guilt.<br>Information can make employers take responsibility.<br>Information can lead to changes in society.<br>Low awareness in society, information is needed.<br>Information enable prevention.	But I think so, that it can be difficult, but still, I think in any case that the information about what you can do in the future is good. That you can change something in your lifestyle.
			It is good to find out in time, so you avoid this.
			Yes, I think it's fantastic even because this would also generate that maybe employers and others have to take on another responsibility and... Because the whole life cycle is one, a squirrel wheel. Many people don't move because they think they don't have time because they work a lot and that should be it, and that and that. So we simply need to pinpoint this. No, I think it's great. Lift it up. It's the basic stuff we neglect. And then we think we'll go and buy a lot of vitamins and we'll live until we're a hundred, even if I sit still and maybe smoke and drink a lot. So that, our society today is really so backwards. Yes. So I think it's great, I think it should be raised much much more and early.
	5.2 Reduce stigma and provide hope	Bowel cancer is ugly cancer.<br>Increased awareness of bowel cancer can reduce stigma.<br>Lift that there is hope, things to do.	It lives a bit in the cloud mouth, bowel cancer. Yes, it is not as popular and even if you find some connections, it is not as much as... big headlines, like this with skin cancer and lung cancer and some others that you then... they get much bigger headlines. Other patient said that that bowel cancer is an

			ugly cancer, you don't want to talk about it.
			-And this is also a form of cancer that when you get your diagnosis, it's far from over..<br>-No, but exactly.<br>-You can, as it were, if you are alert to symptoms, if you are alert to how you live, that you go and check yourself. It's different in different regions if you're called for screening or not and stuff like that, but you can actually do quite a lot in advance, so it's also something that... you could take to heart.<br>-Yes, exactly, because cancer for many is associated with death, you die, and you can actually live a fairly normal life, as a cancer or former cancer patient if you are then allowed to go for checks like this and even the healthcare system meets a and does its job then, which I suppose is quite difficult with everything going on, but for me it has worked great.
